# Long-term risk for incident cancer in patients undergoing coronary artery bypass grafting with or without cardiopulmonary bypass: a nationwide population-based study

**DOI:** 10.1093/ejcts/ezaf110

**Published:** 2025-03-24

**Authors:** Ari Mennander, Susanne J Nielsen, Tanja Skyttä, Maya Landenhed Smith, Andreas Martinsson, Aldina Pivodic, Emma C Hansson, Anders Jeppsson

**Affiliations:** Faculty of Medicine and Health Technology, Tampere, University, Tampere, Finland; Department of Cardiothoracic Surgery, Tampere University Hospital, Heart Hospital, Finland; Department of Molecular and Clinical Medicine, Institute of Medicine, Sahlgrenska Academy, University of Gothenburg, Gothenburg, Sweden; Department of Cardiothoracic Surgery, Sahlgrenska University Hospital, Gothenburg, Sweden; Faculty of Medicine and Health Technology, Tampere, University, Tampere, Finland; Department of Oncology, Tampere University Hospital, Tampere, Finland; Department of Molecular and Clinical Medicine, Institute of Medicine, Sahlgrenska Academy, University of Gothenburg, Gothenburg, Sweden; Department of Cardiothoracic Surgery, Sahlgrenska University Hospital, Gothenburg, Sweden; Department of Molecular and Clinical Medicine, Institute of Medicine, Sahlgrenska Academy, University of Gothenburg, Gothenburg, Sweden; Department of Cardiology, Sahlgrenska University Hospital, Gothenburg, Sweden; APNC AB, Mölndal, Sweden; Department of Molecular and Clinical Medicine, Institute of Medicine, Sahlgrenska Academy, University of Gothenburg, Gothenburg, Sweden; Department of Cardiothoracic Surgery, Sahlgrenska University Hospital, Gothenburg, Sweden; Department of Molecular and Clinical Medicine, Institute of Medicine, Sahlgrenska Academy, University of Gothenburg, Gothenburg, Sweden; Department of Cardiothoracic Surgery, Sahlgrenska University Hospital, Gothenburg, Sweden

**Keywords:** Coronary artery bypass grafting, Incident cancer, Survival

## Abstract

**OBJECTIVES:**

It has been suggested that long-term risk for incident cancer is increased in patients operated with cardiopulmonary bypass. We compared the risk for incident cancer and cancer-specific death between patients undergoing coronary artery bypass grafting (CABG) with and without cardiopulmonary bypass.

**METHODS:**

All patients without a history of cancer undergoing first-time CABG in Sweden during 1997–2020 were included in a nationwide population-based observational cohort study. Individual patient data from the SWEDEHEART registry and 4 other mandatory national registries were merged. The incidence of new cancer was compared between patients operated with or without cardiopulmonary bypass using multivariable Cox proportional hazards regression models adjusted for baseline characteristics, co-morbidities, socioeconomic factors and time of surgery. A propensity score-matched analysis with 3735 well-balanced pairs was also performed.

**RESULTS:**

A total of 81 097 patients undergoing CABG with (*n* = 77 345) and without cardiopulmonary bypass (*n* = 3752) were included. Median follow-up was 8.2 (interquartile range 4.0–13.2) years. The crude event rates were 2.71 and 2.68 per 100 person-years in the patients operated with and without cardiopulmonary bypass, respectively. There was no difference in the adjusted risk for cancer between the groups [adjusted hazard ratio 0.95 (95% confidence interval; CI 0.90–1.01)] or in the risk for cancer-specific death between the groups [adjusted hazard ratio 0.99 (95% CI 0.89–1.09)]. The propensity score-matched analysis showed similar results [hazard ratio 0.96 (95% CI 0.89–1.04) and 0.99 (95% CI 0.85–1.13)], respectively.

**CONCLUSIONS:**

Cardiopulmonary bypass is not associated with an increased risk of incident cancer or cancer-specific mortality in patients undergoing CABG.

## Graphical abstract

**Figure:**
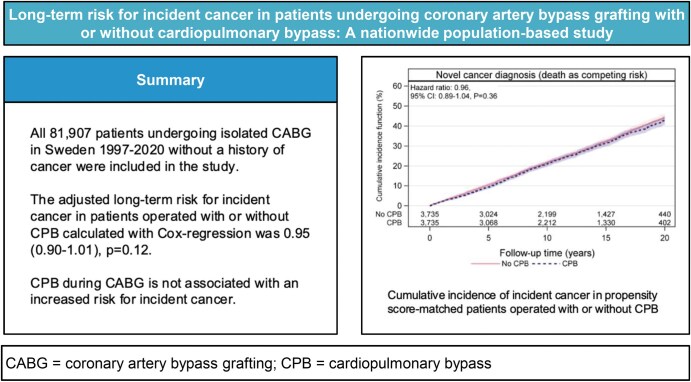

## INTRODUCTION

Coronary artery bypass grafting (CABG) offers a means to treat complex coronary artery disease. CABG can be performed both with and without the use of cardiopulmonary bypass (CPB). Currently, CABG is most often performed with CPB, which is considered technically less demanding [1]. Decision-making to use CPB or not during CABG has remained at the discretion of the surgeon. Still, a few arguments against the use of CPB during CABG remain. Among these is the profound systemic inflammation, which occurs after cardiac surgery with CPB [2, 3]. It has been suggested that the inflammation after cardiac surgery may elicit an immunologic imbalance resulting in an increased long-term risk of incident cancer [3–5]. Accordingly, there has been 1 previous study showing an association between the use of CPB and the risk of incident cancer in patients undergoing CABG [6]. No evidence has been published that contradicts this report. A perceived risk of increased cancer risk with CPB might affect decision-making in patients with coronary artery disease, which in turn could influence outcome after CABG.

We have recently shown in a large nationwide population-based cohort study that patients undergoing cardiac surgery in general do not have an increased long-term risk for cancer as compared to age- and sex-matched individuals from the general population [7]. However, these results do not exclude that there might be differences in incident cancer risk within the population of patients undergoing cardiac surgery due to the use of CPB. We therefore designed an observational cohort study to test the hypothesis that patients undergoing CABG with CPB have an increased risk for incident cancer and cancer-specific mortality compared to patients operated without CPB.

## MATERIALS AND METHODS

### Ethics

The study was approved by the Swedish Ethical Review Authority (registration number 2021-00122) on 31 March 2021. The need for individual patient consent was waived by the Authority. The study was performed in accordance with the Declaration of Helsinki. The manuscript was written in accordance with the Strengthening the Reporting of Observational Studies in Epidemiology (STROBE) statement [8].

### Study population

**Figure 1:**
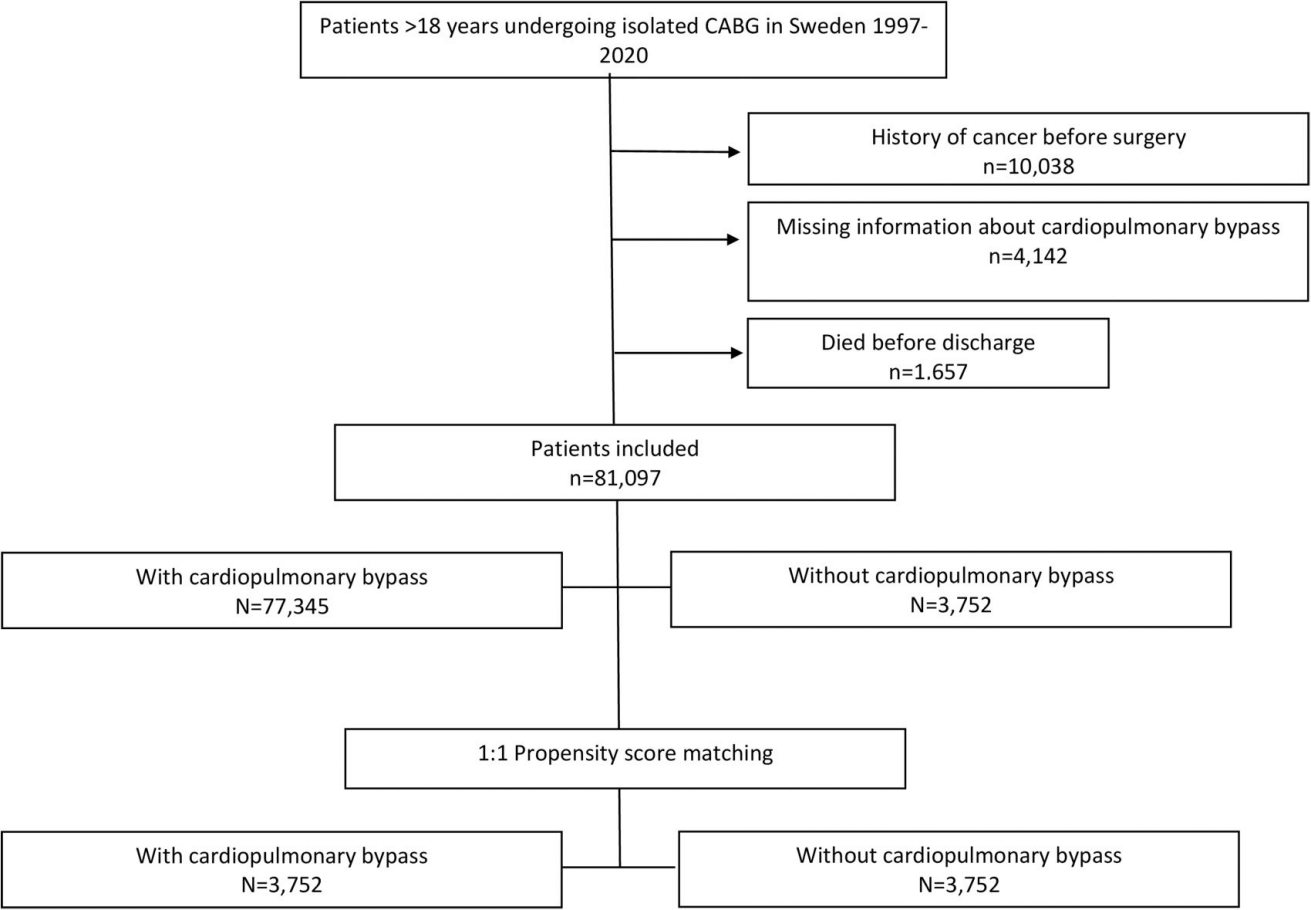
Flowchart of included and excluded patients. CABG: coronary artery bypass grafting.

All patients over 18 years of age undergoing isolated first-time CABG with or without CPB in Sweden from 1997 to 2020 were included. The study population was identified in the Swedish Cardiac Surgery Registry [9], which is part of the SWEDEHEART registry [10]. Patients with a history of cancer at the time of CABG were excluded. A flowchart of included and excluded patients is presented in Fig. 1. The patients were followed from the date of discharge until 31 December 2020 or until death or emigration. Individuals who emigrated during the follow-up period were censored at the time of emigration.

### Study design and data sources

Study end point was time to 1st incident cancer diagnosis after CABG. Individual patient data were collected from 5 nationwide registries and databases with mandatory reporting. The registers were linked via the unique personal identification number given to all Swedish citizens at birth or at immigration [11]. Information about surgical details at baseline, including whether CPB was used or not, was collected from the Swedish Cardiac Registry [10]. The registry contains information on all patients who have undergone cardiac surgery in Sweden since 1992. Additional information about comorbidities and events during the follow-up period was collected from the National Patient Registry (NPR) [12], which contains International Classification of Diseases (ICD)-coded diagnoses for all hospitalizations and outpatient visits in Swedish hospitals since 1987. The ICD codes used for comorbidities and cancers are presented in Supplementary Material, Tables S1 and S2. Diagnoses for comorbidities were collected from 1987 until the date of admission for surgery. Mortality data were collected from the national Cause of Death Register, which has information (in ICD codes) on the date and cause of death. The Swedish Population Register was used for basic demographic information, including data on emigration. The Longitudinal Integration Database for Health Insurance and Labor Market Studies register (LISA) was used to obtain data on socioeconomic factors [13]. Marital status included being married/cohabitating, unmarried/not cohabitating, divorced or widowed; education was stratified into <10 years (compulsory school only), 10–12 years (upper school) and >12 years (college/university level). Annual disposable household income was stratified into quintiles. The Swedish Consumer Price Index was used to adjust income over time.

### Statistical analysis

Continuous variables are described by mean and standard deviation, and median, minimum and maximum, and categorical variables in numbers and percentages. For comparison between 2 groups, Fisher’s exact test was used for dichotomous variables, Chi-squared test for unordered categorical variables, Mantel–Haenszel chi-square trend test for ordered categorical variables and Mann–Whitney *U*-test for continuous variables. Event rates were calculated as the number of events divided by the total number of follow-up years, expressed by 100 person-years. Exact Poisson limits were used to calculate 95% confidence intervals (CIs). Follow-up time was calculated as the time to an event, death, emigration or end of follow-up.

Time to incident diagnosis of cancer and cancer-specific mortality analyses comparing patients undergoing CABG with versus without CPB were performed using Cox proportional hazards models adjusted for age and sex in 1 model, and fully adjusted for age, sex, hypertension, peripheral arterial disease, diabetes, atrial fibrillation, hyperlipidaemia, previous stroke, previous transient ischaemic attack, renal failure, congenital heart disease, chronic respiratory disease, asthma, surgical priority, myocardial infarction and heart failure, as well as marital status, education, income and year of surgery in a 2nd model. The assumption of proportional hazards was checked by visually inspecting the survival curves.

Additionally, for robustness, all analyses were repeated on propensity score 1:1-matched groups including all variables as in the adjustment above. As a matching algorithm, a 1:1 nearest neighbour matching with an optimal calliper width of 0.01 of the standard deviation of the logit of the propensity score was used. Distributions of propensity scores before and after matching as well as standardized mean differences were presented evaluating the goodness of the matching procedure (Supplementary Material, Fig. S1). Cumulative cancer incidence and cancer mortality were described using Nelson–Aalen estimator for patients undergoing CABG with versus without CPB. All tests were two-tailed. *P*-values are presented for descriptive purposes, but because of the large datasets included in the analyses, the interpretation was based on the descriptive data, parameter estimates and their 95% CIs. All analyses were performed by using SAS^®^ Software v9.4 (SAS Institute Inc., Cary, NC, USA).

## RESULTS

### Patient characteristics

**Figure 2:**
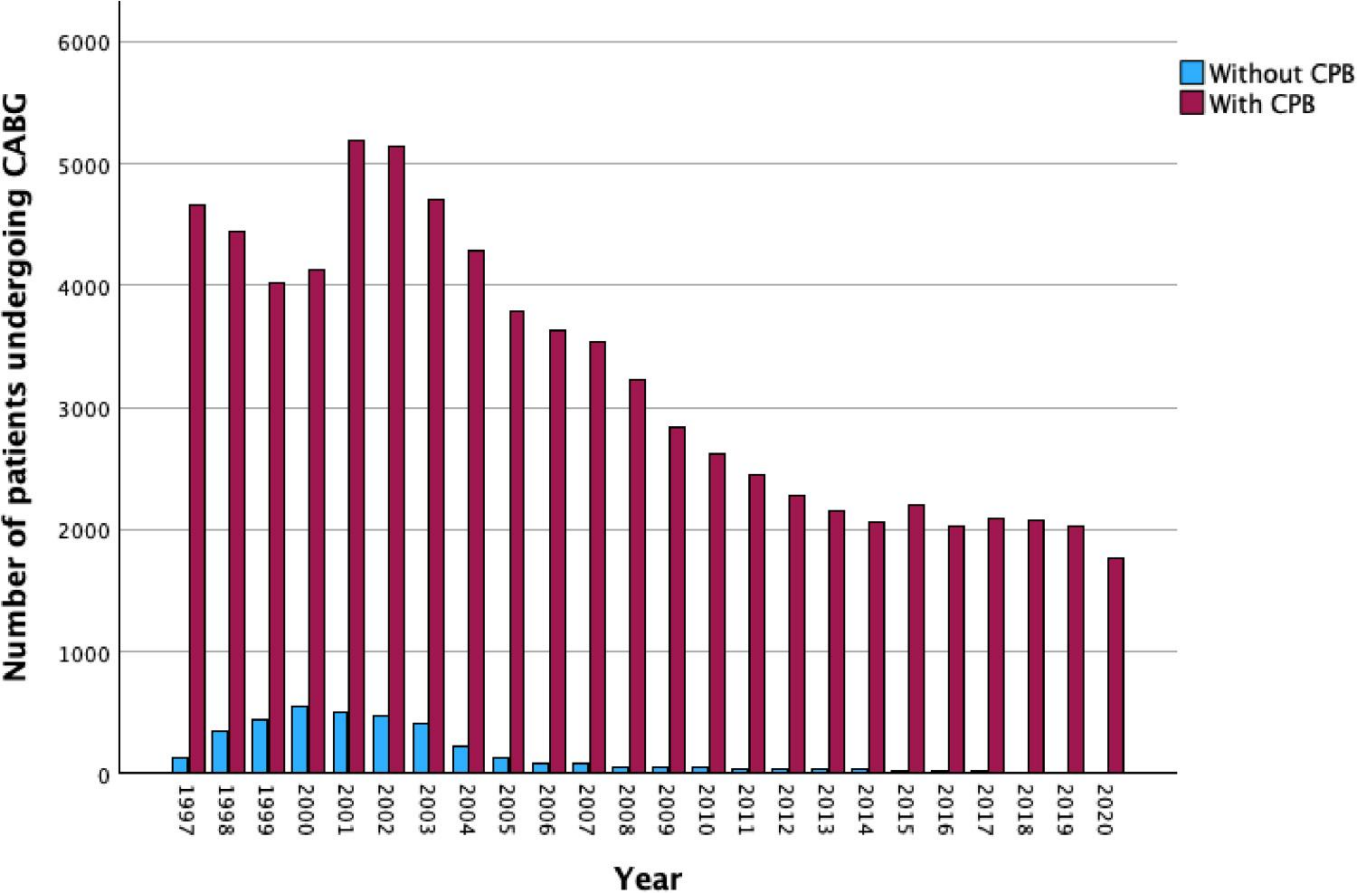
Number of patients undergoing CABG with and without CPB during 1997–2020. CABG: coronary artery bypass grafting; CPB: cardiopulmonary bypass.

Patient demographics are shown in Table 1. Initially, 96 936 patients who underwent CABG in Sweden from 1997 to 2020 were identified, after which 10 038 patients were excluded because of a history of cancer before CABG. Also, patients with missing information about CPB and patients who died before discharge after CABG were excluded. The final study population consisted of 81 907 patients, 77 345 patients operated with CPB and 3752 patients operated without CPB (Fig. 1). There was an annual increase of operations without CPB until the year 2000, after which the number of CABG without CPB steadily declined (Fig. 2).

**Table 1:** Characteristics of patients undergoing cardiac surgery with versus without cardiopulmonary bypass (CPB)

Variable	Without CPB, *N* = 3752	With CPB, *N* = 77 345	*P*-value
Sex			<0.0001
Male	2790 (74.4%)	61 435 (79.4%)	
Female	962 (25.6%)	15 910 (20.6%)	
Age (years)	65.1 (10.1)	66.5 (9.2)	<0.0001
Age category (years)			<0.0001
<65	1699 (45.3%)	30 308 (39.2%)	
65–74	1312 (35.0%)	30 299 (39.2%)	
≥75	741 (19.7%)	16 738 (21.6%)	
Hypertension	1239 (33.0%)	34 009 (44.0%)	<0.0001
Peripheral arterial disease	277 (7.4%)	5518 (7.1%)	0.56
Diabetes mellitus	687 (18.3%)	18 488 (23.9%)	<0.0001
Atrial fibrillation	277 (7.4%)	6145 (7.9%)	0.21
Hyperlipidaemia	995 (26.5%)	22 099 (28.6%)	0.0065
Previous stroke	253 (6.7%)	4561 (5.9%)	0.032
Previous TIA	208 (5.5%)	3681 (4.8%)	0.028
Renal failure	83 (2.2%)	1678 (2.2%)	0.86
Congenital heart disease	6 (0.2%)	218 (0.3%)	0.16
Chronic respiratory disease	221 (5.9%)	4838 (6.3%)	0.37
Asthma	89 (2.4%)	2087 (2.7%)	0.23
Angina pectoris	2486 (66.3%)	43 590 (56.4%)	<0.0001
Previous myocardial infarction	1678 (44.7%)	37 915 (49.0%)	<0.0001
Heart failure	507 (13.5%)	10 358 (13.4%)	0.83
Marital status			0.42
Married/cohabitating	2374 (63.3%)	48 524 (62.7%)	
Not married	388 (10.3%)	9083 (11.7%)	
Divorced	603 (16.1%)	12 612 (16.3%)	
Widowed	386 (10.3%)	7120 (9.2%)	
Missing	1	6	
Education level			0.0040
<10 years	1652 (44.5%)	32 478 (42.6%)	
10–12 years	1462 (39.4%)	30 268 (39.7%)	
>12 years	596 (16.1%)	13 492 (17.7%)	
Missing	42	1107	
Income			<0.0001
Q1 (lowest)	691 (18.4%)	13 397 (17.3%)	
Q2	881 (23.5%)	16 381 (21.2%)	
Q3	865 (23.1%)	17 498 (22.6%)	
Q4	826 (22.0%)	16 746 (21.7%)	
Q5 (highest)	488 (13.0%)	13 317 (17.2%)	
Missing	1	6	

Data are presented as mean±standard deviation, median (range) and number of observations, or number (percentage). For tests between 2 groups with respect to dichotomous variables, Fisher’s exact test was used; Chi-square for unordered categorical variables, Mantel–Haenszel Chi-square trend test for ordered categorical variables and for continuous variables, Mann–Whitney *U*-test.

Q: quintile; TIA: transient ischaemic attack.

Patients operated with CPB were slightly older than those without CPB [(66.5 (9.2) vs 65.1 (10.1) years, respectively)]. Patients who underwent surgery with CPB were more likely to be males and to have a history of hypertension, diabetes mellitus and myocardial infarction, as well as higher levels of education, and higher income than patients undergoing CABG without CPB. In contrast, patients with CPB had less strokes, transient ischaemic attacks, percutaneous coronary interventions and angina pectoris prior to CABG as compared to those without CPB. Median follow-up time was 8.9 years (interquartile range 4.4–13.9) in patients with CPB, and 12.2 years (interquartile range 6.4–17.5) in those without CPB.

### Incident cancer

Numbers of events and event rates overall and for different age groups are presented in Table 2. The overall event rate of incident cancer was 2.71 (95% CI 2.67–2.75) per 100 person-years in patients with CPB, and 2.68 (95% CI 2.53–2.83) per 100 person-years in patients without CPB. The event rate of incident cancer increased by age, both in patients with and without CPB. The different cancer localizations are reported in Table 3. There were no significant differences in cancer localizations between patients operated with or without CPB.

**Table 2:** Event rates, number of events, follow-up time, age and sex adjusted, and fully adjusted hazard ratios (HRs) for novel cancer diagnosis

	No CPB			CPB			CPB vs no CPB	
	*n*/*N* (%) events No. events/follow-up time	Follow-up time, median (IQR)	Event rate (95% CI) per 100 person-years	*n* (%) events No. events/follow-up time	Follow-up time, median (IQR)	Event rate (95% CI) per 100 person-years	Hazard ratio (95% CI)*P-value	Hazard ratio (95% CI)**P-value
All ages	1191/3752 (31.7%)	12.20 (6.35–17.53) Sum = 44 482	2.68 (2.53–2.83)	19 815/77 345 (25.6%)	8.90 (4.41–13.88) Sum = 731 014	2.71 (2.67–2.75)	0.96 (0.91–1.02) *P* = 0.17	0.95 (0.90–1.01) *P* = 0.12
Age category <65 years	460/1699 (27.1%)	16.24 (9.63–19.34) Sum = 24 442	1.88 (1.71–2.06)	6336/30 308 (20.9%)	11.39 (5.90–16.47) Sum = 341879	1.85 (1.81–1.90)	0.98 (0.90–1.08) *P* = 0.75	0.98 (0.89–1.08) *P* = 0.64
Age category 65–74 years	478/1312 (36.4%)	11.05 (5.97–15.90) Sum = 14 248	3.35 (3.06–3.67)	8680/30 299 (28.6%)	8.32 (4.07–13.02) Sum = 268 759	3.23 (3.16–3.30)	0.97 (0.88–1.06) *P* = 0.46	0.96 (0.87–1.05) *P* = 0.36
Age category ≥75 years	253/741 (34.1%)	7.36 (3.71–11.00) Sum = 5792	4.37 (3.85–4.94)	4799/16 738 (28.7%)	6.65 (3.33–10.48) Sum = 120376	3.99 (3.87–4.10)	0.90 (0.79–1.02) *P* = 0.09	0.89 (0.79–1.01) *P* = 0.08

Confidence interval for unadjusted event rates per 100 person-years are obtained from exact Poisson confidence limits. Cox regression was used for time to first event presented by HR.

* Adjusted for age, sex.

** Adjusted for age, sex, hypertension, peripheral arterial disease, diabetes, atrial fibrillation, hyperlipidaemia, stroke, TIA, renal failure, congenital heart disease, chronic respiratory disease, asthma, myocardial infarction, heart failure, marital status, education, income, year of surgery.

CPB: cardiopulmonary bypass; IQR: interquartile range; TIA: transient ischaemic attack.

**Table 3:** Cancer location among patients with and without cardiopulmonary bypass (CPB)

Variable	Total, *N* = 21 006	Without CPB, *N* = 1191	With CPB, *N* = 19 815
Cancer location			
Oral	258 (1.2%)	7 (0.6%)	251 (1.3%)
Digestive	3088 (14.7%)	170 (14.3%)	2918 (14.7%)
Respiratory system	1391 (6.6%)	78 (6.5%)	1313 (6.6%)
Bone, cartilage	27 (0.1%)	2 (0.2%)	25 (0.1%)
Skin	5777 (27.5%)	337 (28.3%)	5440 (27.5%)
Mesothelial, soft tissue	189 (0.9%)	10 (0.8%)	179 (0.9%)
Breast	504 (2.4%)	34 (2.9%)	470 (2.4%)
Female genital system	283 (1.3%)	21 (1.8%)	262 (1.3%)
Male genital system	4615 (22.0%)	252 (21.2%)	4363 (22.0%)
Urinary system	1677 (8.0%)	93 (7.8%)	1584 (8.0%)
Central nervous system	184 (0.9%)	12 (1.0%)	172 (0.9%)
Endocrine system	49 (0.2%)	1 (0.1%)	48 (0.2%)
Unspecified location	1633 (7.8%)	87 (7.3%)	1546 (7.8%)
Lymphoid, haematopoetic	1329 (6.3%)	86 (7.2%)	1243 (6.3%)
More than 1 tumour with different origin	2 (0.0%)	1 (0.1%)	1 (0.0%)

Data are presented as number (percentage).

Age- and sex-adjusted hazard ratios (HR), and multi-adjusted HRs of incident cancers overall and in different age groups according to the multivariable Cox regression, are presented in Table 2. There were no differences in the multi-adjusted long-term risk for incident cancer in patients with CPB as compared with patients without CPB, neither overall [adjusted HR (aHR) 0.95 (0.90–1.01)] nor in the different age groups.

The event rate of cancer-specific death was 0.77 (95% CI 0.75–0.79) per 100 patient years in the CPB group and 0.76 (95% CI 0.69–0.84) per 100 patient years in the no-CPB group. There was no significant difference in the risk for cancer-specific death between the groups [aHR 0.99 (95% CI 0.89–1.09)].

### Propensity score-matched analysis

**Figure 3:**
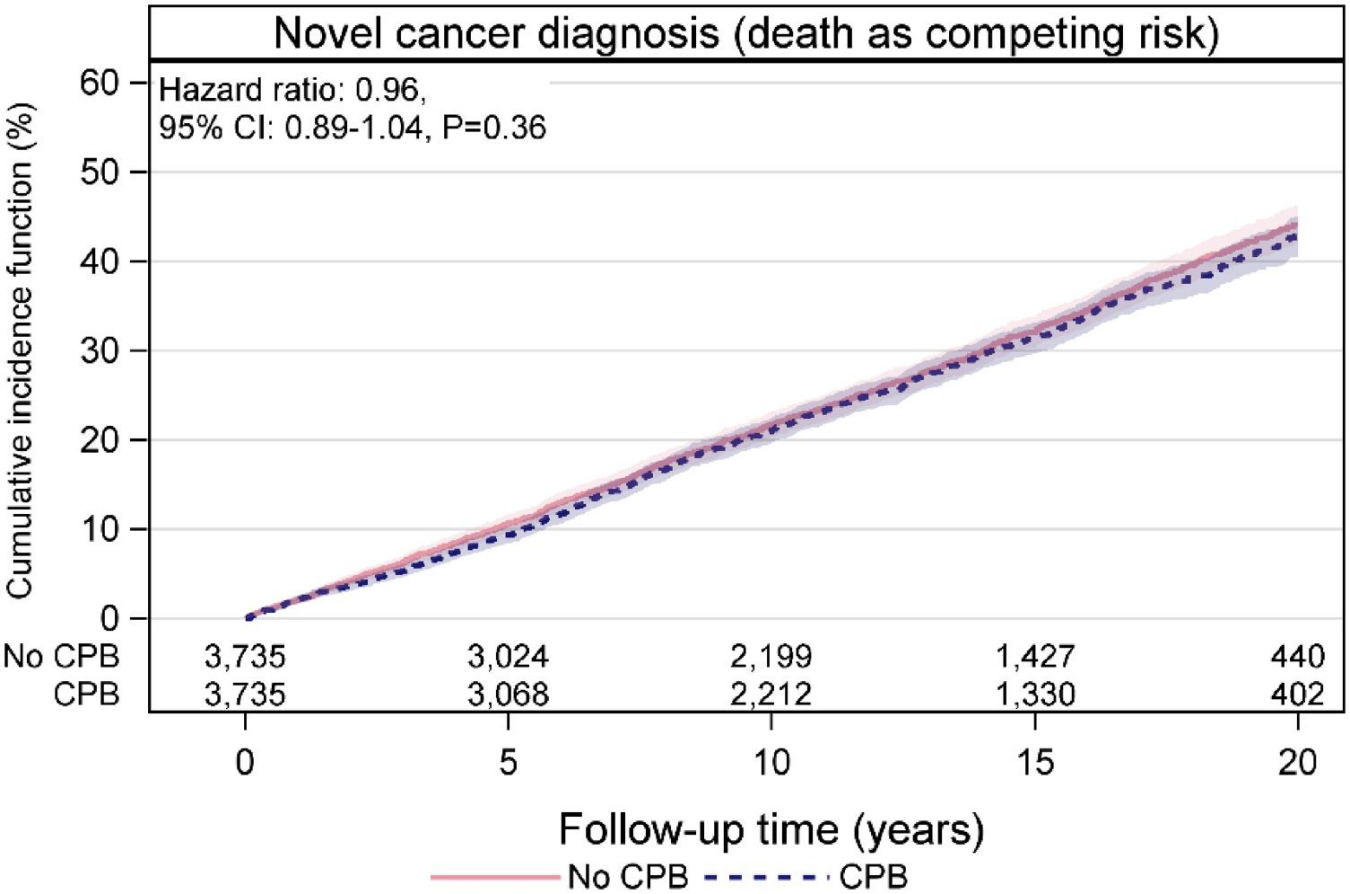
Cumulative incidence of cancer with competing risk of death in patients undergoing CABG with versus without CPB (1:1 propensity score-matched cohort). Shadowed areas represent hazard ratios (HRs) with 95% confidence intervals (CIs). CABG: coronary artery bypass grafting; CPB: cardiopulmonary bypass.

Altogether 3735 1:1-matched pairs of patients with and without CPB were included in the propensity score-matched analysis. The balance of the groups before and after matching is shown in Supplementary Material, Table S3 and Supplementary Material, Fig. S1. Number of events, events rates, age- and sex-adjusted HRs and multi-adjusted HRs of incident cancers overall and in different age groups are presented in Table 4. The crude event rate of incident cancer was 2.57 (95% CI 2.42–2.72) per 100 person-years in patients with CPB, and 2.68 (95% CI 2.53–2.84) per 100 person-years in patients without CPB. The event rate of incident cancer increased by age, both in patients with and without CPB. There were no differences in long-term risk for incident cancer in the propensity score-matched population in both patient groups, neither overall [aHR 0.96 (95% CI 0.89–1.04)] nor in the different age groups. The cumulative incidence of cancer with competing risk of death is shown in Supplementary Material, Fig. S2 and in the propensity score-matched population in Fig. 3. There was no significant difference in the risk for cancer-specific death between the propensity-matched groups [aHR 0.98 (95% CI 0.85–1.13)].

**Table 4:** Event rates, number of events, follow-up time and HRs (Cox regression) for novel cancer diagnosis comparing patients with versus without cardiopulmonary bypass (CPB) (propensity score 1:1-matched groups)

	No CPB			CPB			CPB vs No CPB
	*n*/*N* (%) events No. events/follow-up time	Follow-up time, median (IQR)	Event rate (95% CI) per 100 person-years	*n* (%) events No. events/follow-up time	Follow-up time, median (IQR)	Event rate (95% CI) per 100 person-years	Hazard ratio (95% CI) *P*-value
All ages	1186/3735 (31.8%)	12.19 (6.36–17.52) Sum = 44 269	2.68 (2.53–2.84)	1131/3735 (30.3%)	12.04 (6.53–17.15) Sum = 44 056	2.57 (2.42–2.72)	0.96 (0.89–1.04) *P* = 0.36
Age category <65 years	458/1688 (27.1%)	16.23 (9.65–19.31) Sum = 24 268	1.89 (1.72–2.07)	447/1688 (26.5%)	15.64 (9.76–19.21) Sum = 24 193	1.85 (1.68–2.03)	0.99 (0.87–1.13) *P* = 0.85
Age category 65–74 years	477/1310 (36.4%)	11.05 (5.97–15.90) Sum = 14 230	3.35 (3.06–3.67)	451/1310 (34.4%)	10.77 (5.73–15.65) Sum = 14 003	3.22 (2.93–3.53)	0.96 (0.85–1.10) *P* = 0.57
Age category ≥75 years	251/737 (34.1%)	7.39 (3.72–11.00) Sum = 5771	4.35 (3.83–4.92)	233/737 (31.6%)	7.60 (4.25–11.36) Sum = 5860	3.98 (3.48–4.52)	0.92 (0.77–1.10) *P* = 0.36

Confidence interval for unadjusted event rates per 100 person-years are obtained from exact Poisson confidence limits. Cox regression was used for time to first event presented by HR.

IQR: interquartile range.

## DISCUSSION

The main finding of this large population-based nationwide study was that patients undergoing CABG with or without CPB have similar adjusted long-term risk for incident cancer and cancer-specific mortality.

Three previous studies indicate that adult cardiac surgery patients have an increased risk of developing cancer [4–6]. Alameddine *et al.* [4] reported that patients undergoing cardiac surgery have a 3-fold increase in the 5-year cumulative incidence of cancer, as compared with the incidence in the general population. Vieira *et al.* [5] reported from the MASS II study that patients with coronary artery disease undergoing CABG had the highest incidence of non-cardiac causes of death and, specifically, a higher tendency towards cancer-related deaths, compared to patients treated with percutaneous coronary intervention or medical treatment. Furthermore, and of more relevance to the present study, Pinto *et al.* [6] found a 17% increase in cancer incidence in 43 347 patients undergoing CABG with CPB, compared to CABG without CPB. Based on these studies, we hypothesized that patients undergoing CABG with CPB have an increased long-term risk for incident cancer.

Contrary to our hypothesis, we found no difference in incident cancer risk between patients operated with and without CPB in this large cohort study. The discrepancy between the present and earlier studies may be explained by the differences in study population and follow-up time. Pinto *et al.* [6] included 43 347 patients, out of which 29.7% were operated without CPB. The corresponding figures in the present study were 81 907 and 3.5%, respectively. Furthermore, and potentially more importantly, Pinto *et al*.’s follow-up time was restricted to a maximum of 8 years, while it was up to 24 years in the present study. There are also other methodological differences in definitions and statistical approaches that may contribute to the diverging results.

Our results strongly argue against the idea that the use of CPB would influence the risk for incident cancer and cancer-specific mortality. Further evidence against an increased cancer risk in cardiac surgery patients comes from our recent study where we compared the risk for incident cancer and cancer-specific mortality in cardiac surgery patients with the risk in age- and sex-matched individuals from the general population [7]. In that study, patients undergoing cardiac surgery had lower risk for incident cancer and cancer-specific mortality than control subjects. Furthermore, in another study, no differences in cancer recurrence and cancer-related deaths were detected in patients operated with and without CPB among cardiac surgery patients with cancer present at the time of surgery [14]. Taken together, these studies refute the theory that cardiac surgery, with and without CPB, induces an increased cancer risk, and should hence not influence decision-making in patients with coronary artery disease.

The studies supporting a link between cardiac surgery and incident cancer risk speculated that the pronounced postoperative inflammatory response was related to a temporary down-regulation of immunomodulation during surgery with CPB [4–6]. A link between inflammation, cardiovascular disease and cancer has previously been demonstrated [15, 16]. However, the importance of CPB for the magnitude and impact of the inflammatory response after cardiac surgery remains unsettled. In a study comparing patients operated with CPB to those without CPB, CPB *per se* elicited a rather modest immune response after CABG [17].

### Limitations

The limitations include those inherent in an observational study, such as unregistered confounders, and treatment bias. Information about smoking is lacking in the database. We did, however, adjust for socioeconomic factors, which are related to smoking habits [18]. The majority of patients operated without CPB were included during the first 5 years of the study period. The frequency of CABG without CPB is low in Sweden and has decreased considerably over time. To adjust for this, we included the year of surgery in the statistical models. Further limitations include the risk of undiagnosed cancer at the time of inclusion. However, there are no indications that the proportion of undiagnosed cancer at the time of surgery would differ between patients operated with and without CPB. The strengths of this study include the real-world setting, the large study cohort with national coverage, the validated data sources [10, 11, 13] and the complete long-term follow-up. Furthermore, the results were statistically robust and independent of the statistical methods.

## CONCLUSION

Patients undergoing CABG with CPB had a similar risk for incident cancer and cancer-specific mortality during long-term follow-up as compared to those operated without CPB.

## Supplementary Material

ezaf110_Supplementary_Data

## Data Availability

The data in this study will be provided upon reasonable request to the corresponding author after approval from the SWEDEHEART registry, the Swedish Ethical Review Authority and the National Board of Health and Welfare.

## ABBREVIATIONS

aHR: Adjusted hazard ratio
CABG: Coronary artery bypass grafting
CI: Confidence interval
CPB: Cardiopulmonary bypass
HR: Hazard ratio
ICD: International Classification of Diseases
LISA: Longitudinal Integration Database for Health Insurance and Labor Market Studies register
NPR: National Patient Registry
STROBE: Strengthening the Reporting of Observational Studies in Epidemiology
