# Supplementary material for: Long-term risk for incident cancer in patients undergoing coronary artery bypass grafting with or without cardiopulmonary bypass: a nationwide population-based study
Source: Eur J Cardiothorac Surg. 2025 Mar 24;67(4):ezaf110. doi: 10.1093/ejcts/ezaf110 (PMC11975279; doi:10.1093/ejcts/ezaf110)

**SUPPLEMENTARY MATERIAL TO**

**Long-term risk for incident cancer in patients undergoing coronary artery bypass grafting with or without cardiopulmonary bypass:** **A nationwide population-based study**

Ari Mennander^a,b^, Susanne J. Nielsen^c,d^, Tanja Skyttä^b,e^, Maya Landenhed Smith^c,d^, Andreas Martinsson^d,f^, Aldina Pivodic^g^, Emma C. Hansson^c,d^, Anders Jeppsson^c,d^

^a^Tampere University Hospital, Heart Hospital, Tampere, Finland; ^b^Faculty of Medicine and Health Technology, Tampere University, Tampere, Finland; ^c^Department of Cardiothoracic Surgery, Sahlgrenska University Hospital, Gothenburg, Sweden; ^d^Department of Molecular and Clinical Medicine, Institute of Medicine, Sahlgrenska Academy, University of Gothenburg, Gothenburg, Sweden; ^e^Department of Oncology, Tampere University Hospital, Tampere, Finland; ^f^Department of Cardiology, Sahlgrenska University Hospital, Gothenburg, Sweden; ^g^APNC AB, Gothenburg, Sweden

**Content**

**Tables**

1. International classification of diseases (ICD) codes used for classification of comorbidities at baseline.
2. International classification of diseases (ICD) codes used for classification of location of cancers.
3. Patient characteristics in patients undergoing CABG with and without CPB (PS 1:1 matched groups).

**Figures**

1. Distribution of propensity scores for patients undergoing CABG with and without CPB before and after matching.
2. Cumulative incidence curve for incident cancer diagnosis adjusted for death as competing risk for patients undergoing CABG with and without CPB.

**Supplementary Table 1.** International classification of diseases (ICD) codes used for classification of comorbidities.

| Comorbidity | ICD9 | ICD10 |
| --- | --- | --- |
|  | **1986-1996** | **1997-2020** |
| Novel cancer | 140-208 | C00-C97 |
| Hypertension | 401-405 | I10.0-I15.9 |
| Peripheral artery disease | 440, 443X, 444, 447 | I70, I73.9, I74, I77 |
| Diabetes mellitus | 250 | E10-E14 |
| Atrial fibrillation | 427D | I48 |
| Hyperlipidemia | 272.0, 272.01, 272.09 | E78 |
| Stroke | 431-434, 436 | I61-I64 |
| Transient ischemic attack | 435, 436 | I65, I66, G45 |
| Renal failure | 584-586 | N17-N19 |
| Chronic pulmonary disease | 490-496 | J40-J47 |
| Previous myocardial infarction | 410 | I21.0-I21.4 |
| Heart failure | 428 | I50, I42-143.8, I11.0, I13.0, I13.2 I50, I25.5 |

**Supplementary Table 2.** International classification of diseases (ICD) codes used for classification of location of cancers.

| Cancer location | ICD9 | ICD10 |
| --- | --- | --- |
|  | **1986-1996** | **1997-2020** |
| Oral | 140-149 | C00-C14 |
| Digestive | 150-159 | C15-C26 |
| Respiratory system | 160-165 | C30-C39 |
| Bone, Cartilage | 170-171 | C40-C41 |
| Skin | 172-173,176 | C43-C44 |
| Mesothelial, soft tissue |  | C45-C49 |
| Breast | 174-175 | C50 |
| Female genital system | 179-184 | C51-C58 |
| Male genital system | 185-187 | C60-C63 |
| Urinary system | 188-189 | C64-C68 |
| Central nervous system | 190-192 | C69-C72 |
| Endocrine system | 193-194,209 | C73-C75 |
| Unspecified location | 195 | C76-C80 |
| Lymphoid, hematopoetic | 200-208 | C81-C96 |
| More than one tumor with different origin |  | C97 |

**Supplementary Table 3**. Patient characteristics in patients with and without cardiopulmonary bypass (CPB) (propensity score 1:1 matched groups).

| **Variable** | **Without CPB N=3735** | **With CPB N=3735** | **p-value** |
| --- | --- | --- | --- |
| Sex |  |  | 1.00 |
| Male | 2784 (74.5%) | 2784 (74.5%) |  |
| Female | 951 (25.5%) | 951 (25.5%) |  |
| Age (years) | 65.1 (10.1) | 65.3 (9.6) | 0.59 |
| Age category (years) |  |  | 1.00 |
| <65 | 1688 (45.2%) | 1688 (45.2%) |  |
| 65-74 | 1310 (35.1%) | 1310 (35.1%) |  |
| ≥75 | 737 (19.7%) | 737 (19.7%) |  |
| Hypertension | 1235 (33.1%) | 1155 (30.9%) | 0.047 |
| Peripheral arterial disease | 272 (7.3%) | 227 (6.1%) | 0.037 |
| Diabetes mellitus | 683 (18.3%) | 648 (17.3%) | 0.29 |
| Atrial fibrillation | 275 (7.4%) | 234 (6.3%) | 0.06 |
| Hyperlipidemia | 990 (26.5%) | 950 (25.4%) | 0.29 |
| Previous stroke | 250 (6.7%) | 204 (5.5%) | 0.026 |
| Previous TIA | 205 (5.5%) | 163 (4.4%) | 0.025 |
| Previous PCI | 571 (15.3%) | 319 (8.5%) | <.0001 |
| Renal failure | 79 (2.1%) | 72 (1.9%) | 0.56 |
| Congenital heart disease | 6 (0.2%) | 5 (0.1%) | 0.76 |
| Chronic respiratory disease | 216 (5.8%) | 183 (4.9%) | 0.09 |
| Asthma | 85 (2.3%) | 73 (2.0%) | 0.33 |
| Angina pectoris | 2472 (66.2%) | 2283 (61.1%) | <.0001 |
| Previous myocardial infarction | 1674 (44.8%) | 1729 (46.3%) | 0.20 |
| Heart failure | 503 (13.5%) | 445 (11.9%) | 0.044 |
| Marital status |  |  | 0.16 |
| Married/cohabitating | 2364 (63.3%) | 2441 (65.4%) |  |
| Not married | 386 (10.3%) | 338 (9.1%) |  |
| Divorced | 599 (16.0%) | 592 (15.9%) |  |
| Widowed | 386 (10.3%) | 363 (9.7%) |  |
| Missing | 0 | 1 |  |
| Education level |  |  | 0.61 |
| <10 years | 1645 (44.5%) | 1685 (45.6%) |  |
| 10-12 years | 1456 (39.4%) | 1408 (38.1%) |  |
| >12 years | 593 (16.1%) | 601 (16.3%) |  |
| Missing | 41 | 41 |  |
| Income |  |  | 0.53 |
| Q1 (lowest) | 689 (18.4%) | 658 (17.6%) |  |
| Q2 | 877 (23.5%) | 867 (23.2%) |  |
| Q3 | 860 (23.0%) | 901 (24.1%) |  |
| Q4 | 821 (22.0%) | 820 (22.0%) |  |
| Q5 (highest) | 488 (13.1%) | 488 (13.1%) |  |
| Missing | 0 | 1 |  |
| Year of surgery |  |  | 0.17 |
| 1997 | 121 (3.2%) | 329 (8.8%) |  |
| 1998 | 341 (9.1%) | 409 (11.0%) |  |
| 1999 | 448 (12.0%) | 393 (10.5%) |  |
| 2000 | 541 (14.5%) | 428 (11.5%) |  |
| 2001 | 503 (13.5%) | 466 (12.5%) |  |
| 2002 | 477 (12.8%) | 403 (10.8%) |  |
| 2003 | 404 (10.8%) | 321 (8.6%) |  |
| 2004 | 217 (5.8%) | 212 (5.7%) |  |
| 2005 | 131 (3.5%) | 157 (4.2%) |  |
| 2006 | 86 (2.3%) | 115 (3.1%) |  |
| 2007 | 92 (2.5%) | 101 (2.7%) |  |
| 2008 | 48 (1.3%) | 80 (2.1%) |  |
| 2009 | 50 (1.3%) | 53 (1.4%) |  |
| 2010 | 51 (1.4%) | 50 (1.3%) |  |
| 2011 | 37 (1.0%) | 32 (0.9%) |  |
| 2012 | 30 (0.8%) | 28 (0.7%) |  |
| 2013 | 31 (0.8%) | 24 (0.6%) |  |
| 2014 | 30 (0.8%) | 21 (0.6%) |  |
| 2015 | 26 (0.7%) | 29 (0.8%) |  |
| 2016 | 19 (0.5%) | 20 (0.5%) |  |
| 2017 | 26 (0.7%) | 18 (0.5%) |  |
| 2018 | 13 (0.3%) | 22 (0.6%) |  |
| 2019 | 8 (0.2%) | 13 (0.3%) |  |
| 2020 | 5 (0.1%) | 11 (0.3%) |  |
| Data are presented as mean±standard deviation, median (range) and number of observations, or number (percentage). For test between two groups with respect to dichotomous variables Fisher's exact test was used, for ordered categorical variables Mantel-Haenszel Chi-square trend test, and for continuous variables Mann-Whitney U-test. | | | |

**Supplementary Figure 1**. Panel A: Distribution of the propensity scores for cases with vs. without CPB before matching.


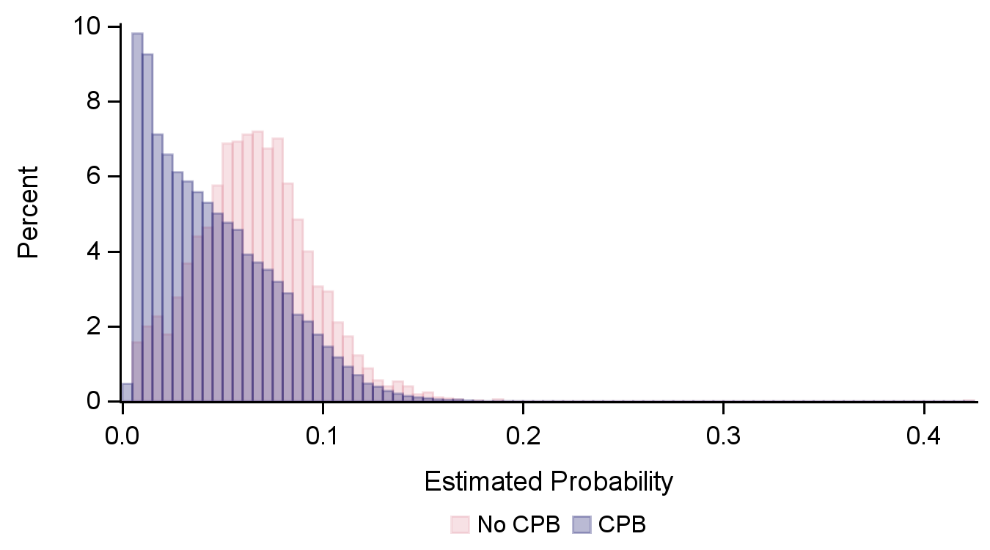


Panel B. Distribution of the propensity scores for cases with vs. without CPB after matching.


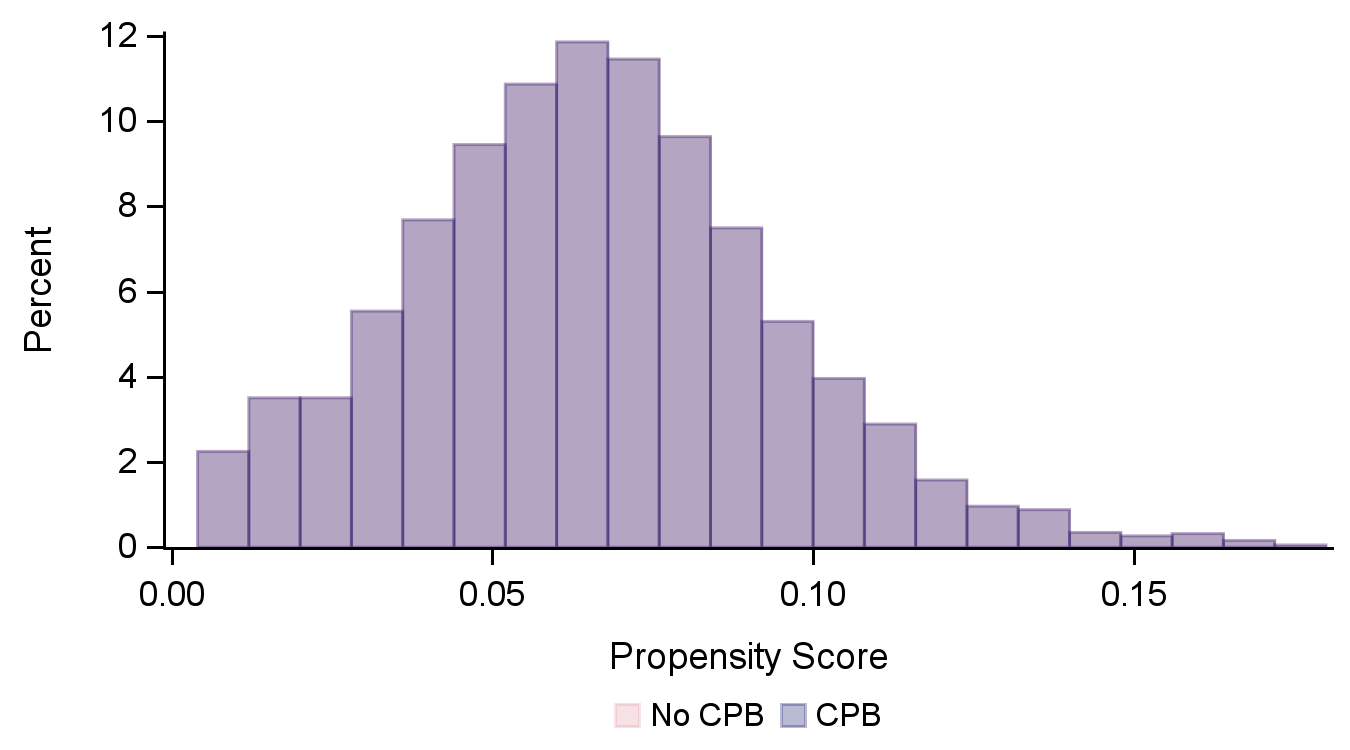


Panel C. Standardized mean differences for variables included in the matching of cases with vs. without CPB, before and after matching.


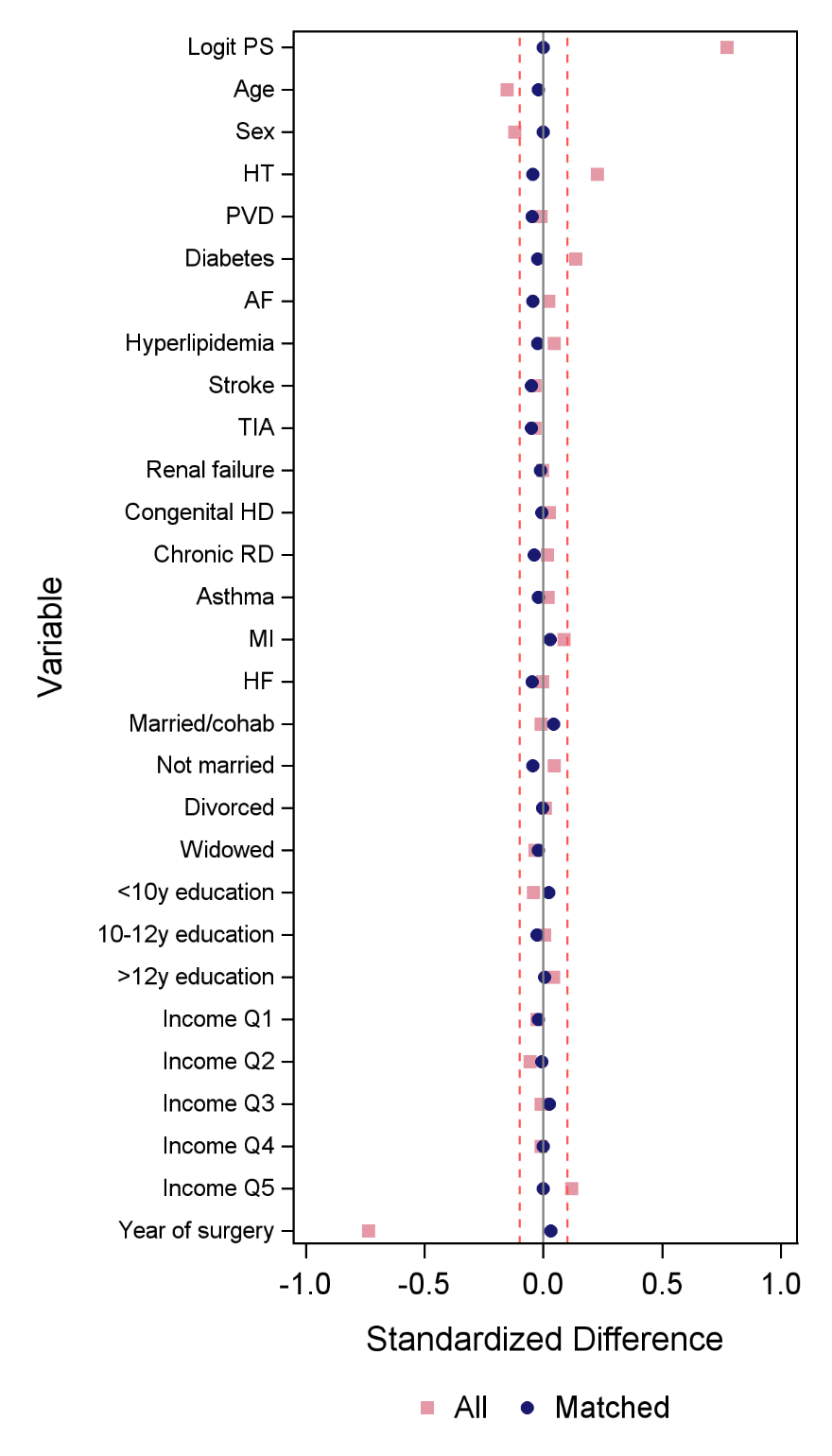


Abbreviations: PS= propensity score, HT= hypertension, PVD= peripheral vascular disease, AF= atrial fibrillation, TIA = transient ischaemic attack, HD= heart disease, RD = respiratory disease, MI = myocardial infarction, HF = heart failure, Q= quartile

**Supplementary Figure 2.** Cumulative incidence curve for novel cancer diagnosis adjusted for death as competing risk for patients undergoing coronary artery grafting with vs without CPB. Shadowed areas represent Hazard Ratios (HRs) with 95% confidence intervals.


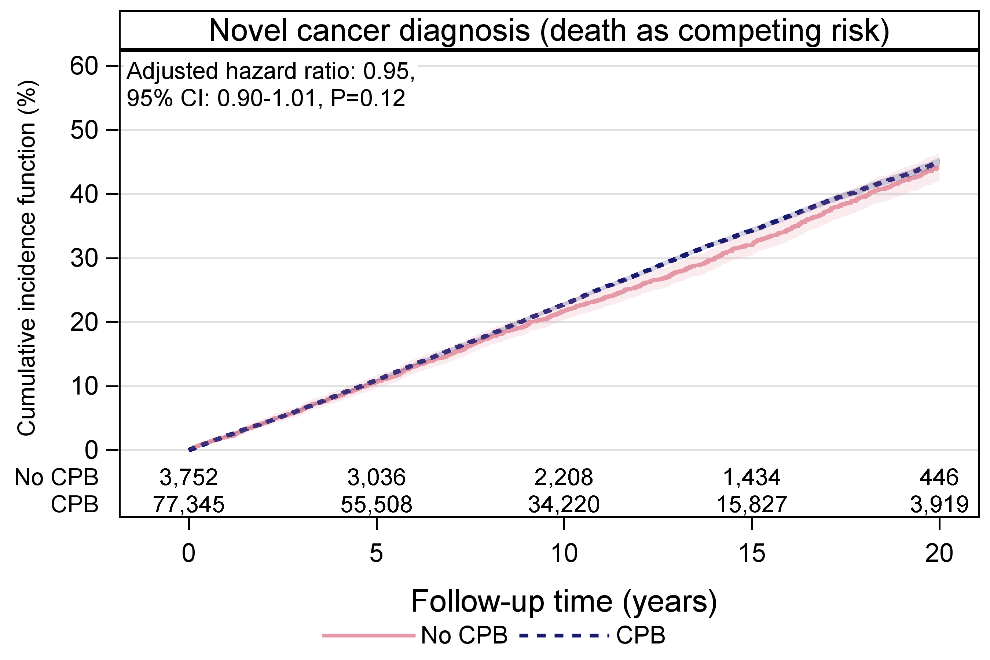

Supplement: ezaf110_Supplementary_Data [file ezaf110_supplementary_data.docx]
